# Aggregation Control by Multi-stimuli-Responsive Poly(*N*-vinylamide) Derivatives in Aqueous System

**DOI:** 10.1186/s11671-017-2221-7

**Published:** 2017-07-21

**Authors:** Ryo Kawatani, Yasuhiro Nishiyama, Hironari Kamikubo, Kiyomi Kakiuchi, Hiroharu Ajiro

**Affiliations:** 1 Graduate School of Materials Science, Nara Institute of Science and Technology, 8916-5, Takayama-cho, Ikoma, Nara 630-0192 Japan; 2 Institute for Research Initiatives, Nara Institute of Science and Technology, 8916-5, Takayama-cho, Ikoma, Nara 630-0192 Japan; 3 JST PRESTO, 4-1-8 Honcho, Kawaguchi, Saitama 332-0012 Japan

**Keywords:** Thermosensitive, Nanoparticle, *N*-Vinylamide, Azobenzene

## Abstract

Thermal and photo responsive copolymer based on *N*-vinylamide backbone was designed. Methoxyethyl group and azobenzene were selected to improve hydrophilicity and photoresponsive moieties, respectively. The *N*-(methoxyethyl)-*N*-vinylformamide was synthesized and copolymerized with *N*-vinylformamide by free radical polymerization. In order to control the nanosized structures, poly(*N*-vinylformamide) derivatives bearing azobenzene at the *N*-position near to the vinyl polymer main chain were synthesized by polymer reaction with the poly(*N*-vinylformamide-*co*-*N*-(methoxyethyl)-*N*-vinylformamide) and azobenzene. Aggregation size of the multi-stimuli-responsive polymer was controlled by preparation of the hydrophobic interaction at around *N*-position.

**Electronic supplementary material:**

The online version of this article (doi:10.1186/s11671-017-2221-7) contains supplementary material, which is available to authorized users.

## Background

In the development of polymer materials, the addition of multi-stimuli-responsive behavior is imperative [1–3], for example, thermo-redox [4], thermo-salt [5], thermo-pH [6–8] among others [9, 10]. The lower critical solution temperature (LCST) behavior is one of the thermosensitive properties that has been investigated intensively [11–16]. Furthermore, some researchers have reported that multi-stimuli-responsive polymers were synthesized from thermosensitive polymers and photoresponsive compounds among others, for example, azobenzene derivatives as photoresponsive compounds [17–22]. Among these, controlled polymer aggregation in water has been achieved [23–28]. For instance, controlled molecular inclusions by supermolecular recognition and controlled drug release have been investigated using multi-stimuli-responsive polymers [29]. Through these examples, aggregation control in water is desirable and needs to be better understood.

Contrastingly, poly(*N*-vinylamide) derivatives have been investigated since the development of the novel synthesis route of poly(*N*-vinylacetamide) (PNVA) was reported [30] Furthermore, simpler synthesis routes to the monomers were developed [31]. Poly(*N*-vinylamide) derivatives have several useful properties. For example, the PNVA is an amphiphilic polymer [32], poly(*N*-vinylisobutylamide) (PNVIBA) is a thermosensitive polymer [33], and poly(*N*-vinylformamide) (PNVF) is a polycation precursor. Furthermore, the PNVF can be hydrolyzed in alkaline conditions and converted to cationic poly(*N*-vinylamine) derivatives without releasing toxic low molecular amine [34]. In addition, poly(*N*-vinylamide) derivatives were stable for a long period against irradiated UV [35]. This motivates this research to use these polymers for possible application as photoresponsive materials. However, research in poly(*N*-vinylamide) derivatives is lacking compared to poly(acrylamide) derivatives which are structural isomers of poly(*N*-vinylamide) derivatives [36, 37]. This is because *N*-vinylamide derivatives are low-reactivity monomers such as unconjugated vinyl monomer [38].

**Fig. 1:**
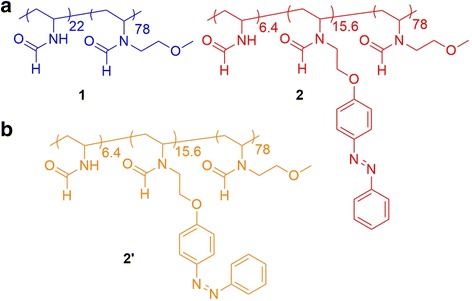
Chemical structures of **1** (**a**) and **2** and **2’** (**b**)

In this study, we synthesized the monomer of *N*-vinylamide derivatives bearing a methoxyethyl group at the *N*-position of NVF (MOENVF: *M*_w_ = 129) in order to improve the hydrophilicity instead of hydrophobic alkyl substituents [39]. In the previous work, we have shown the length and structural isomers of alkyl substituents at the *N*-position of *N*-vinylamide copolymers, resulting in various LCST. Poly(NVF-*co*-MOENVF) (**1**) was synthesized by free radical polymerization, resulting in a hydrophilic polymer (Fig. 1). When **1** had photosensitive hydrophobic units, such as *N*-vinylformamide bearing azobenzene derivatives at the *N*-position (NVFazo), where the *M*_w_ of monomer unit is 295, **1** became a photosensitive and thermosensitive amphipathic polymer depending on its hydrophobicity. Then, poly(NVF-*co*-MOENVF-*co*-NVFazo) (**2**) was synthesized by polymer reaction (Fig. 1). Using the copolymers, nanoparticles based on poly(*N*-vinylamide)s were prepared, and the aggregates were controlled by temperature and UV irradiation.

## Methods

### Materials

*N*-vinylformamide (NVF) was purchased from Tokyo Chemical Industry Co., Ltd. (Japan) and was distilled. Sodium hydride (NaH) 60% in oil, 2-bromoethyl methyl ether, 1, 2-dibromoethane and 4-(phenylazo) phenol, and anhydrous tetrahydrofuran (THF) were purchased from Tokyo Chemical Industry Co., Ltd. (Japan) and used as received. Anhydrous *N,N*-dimethylformamide (DMF), acetone, toluene, ethyl acetate, and magnesium sulfate were purchased from NACALAI TESQUE, INC. (Japan). Azobisisobutyronitrile (AIBN) and hexane were purchased from Wako Pure Chemical Industries Ltd. (Japan). Diethyl ether was purchased from AZBIO CORP. (Japan).

### Polymerization

The procedure of the typical free radical copolymerization of MOENVF and NVF was as follows. MOENVF (1.03 g, 8 mmol), NVF (0.14 g, 2 mmol), toluene (5 ml), and AIBN (0.044 g, 0.25 mmol) were combined in a 50-mL glass tank. The reactor was capped with septa; then, N_2_ bubbling was performed for 2 min. The reaction mixture was heated up to 60 °C to start polymerization. After 24 h, it was cooled down to room temperature, and the reaction mixture was poured into 500 mL of diethyl ether. The polymer was washed twice by diethyl ether and recovered by centrifugation. The polymer poly(NVF-*co*-MOENVF) was obtained (0.69 g, yield 59%) after drying under vacuum at 30 °C for 12 h.

The poly(NVF-*co*-MOENVF) (0.20 g, solid) and NaH (0.4 mmol, 0.011 g, solid) were combined in a 50-mL glass tank. The reactor was capped with septa; then, N_2_ was suffused after which, DMF (1 ml) was slowly added by syringe at 100 °C. After stirring for 15 min at 100 °C, 1-bromoether azobenzene (0.4 mmol, 0.12 g, solid) was slowly added during N_2_ belching. After stirring for 12 h, water (1 ml) was slowly added by syringe at room temperature. Whereafter, DMF (2 ml) was added and poured into 500 mL acetone. The polymer was washed five times by acetone and recovered by centrifugation. The polymer poly(NVF-*co*-MOENVF-*co*-NVFazo) was obtained (0.29 g, yield 43%) after drying under vacuum at 30 °C for 12 h.

### Measurements

Size-exclusion chromatography (SEC) was achieved using a Chem NAV system with polystyrene standards at 40 °C, equipped with AS-2055, CO-2065, PU-2080, UV-2075, and RI-2031 (JASCO Corporation, Japan). TSKgel SuperH4000 and TSKgel GMHXL (Tosoh Corporation, Japan) were connected, and dimethylformamide (DMF) was used as the eluent. Proton nuclear magnetic resonance (^1^H NMR) spectra were measured by JEOL JNM-ECX 400 (400 MHz). Transmittance *vs.* temperature was monitored by UV-2600 (Shimadzu Corporation). UV light (330 nm) was irradiated by MAX-303 with 330-nm filter at 300W Xenon lamp (ASAHI SPECTRA USA Inc.) for 10 min. DLS was measured by ZEN3600 Zetasizer Nano ZS (Malvern Instruments Ltd.) with He-Ne laser at 633 nm.

## Results and Discussion

Firstly, **1** was synthesized by radical polymerization using AIBN. NVF and MOENVF were copolymerized in toluene at 60 °C, and the product was purified by reprecipitation with diethyl ether. Table 1 shows the analytical data for **1** and **2**. The insoluble part was filtered and vacuum dried, and a white powder was obtained at 59% yield (Table 1, run 1). This result was consistent with previous research [39]. **2** was then prepared by the polymer reaction between **1** and 1-bromoethyl azobenzene with NaH in DMF. The photoresponsive azobenzene units were introduced into **1** with monomer synthesis. The product was purified by reprecipitation with acetone at 42% yield (Table 1, run 2). Unit ratios in **1** and **2** were calculated for integral intensity from ^1^H NMR spectra (Additional file 1: Figure S2). Comonomer units in **1** corresponded to the feed ratio of polymerization (NVF:MOENVF = 20:80). As estimated, 72% of NVF units in the copolymer converted to NVAazo, although not all units of NVF reacted (16/22 units were converted).

**Table 1:** Analytical data for copolymers

Run	Copolymer	Yield (%)	Unit ratio in copolymer	*M*_n_^e^ (×10^3^)	PDI^e^
1^a^	**1**	59^c^	NVF:MOENVF = 22:78	8.1	3.8
2^b^	**2**	42^d^	NVF:NVFazo:MOENVF = 6:16:78	4.0	4.8

^a^Polymerization conditions: Solvent = toluene. Conc. = 2 M. Initiator = AIBN. Temp. = 60 °C. Time = 24 h

^b^Polymer reaction conditions: Solvent = DMF. Conc. = 1 M. Temp. = 80 °C. Time = 10 h

^c^Diethyl ether-insoluble part

^d^Acetone-insoluble part

^e^Determined by SEC with polystyrene standard in DMF

**Fig. 2:**
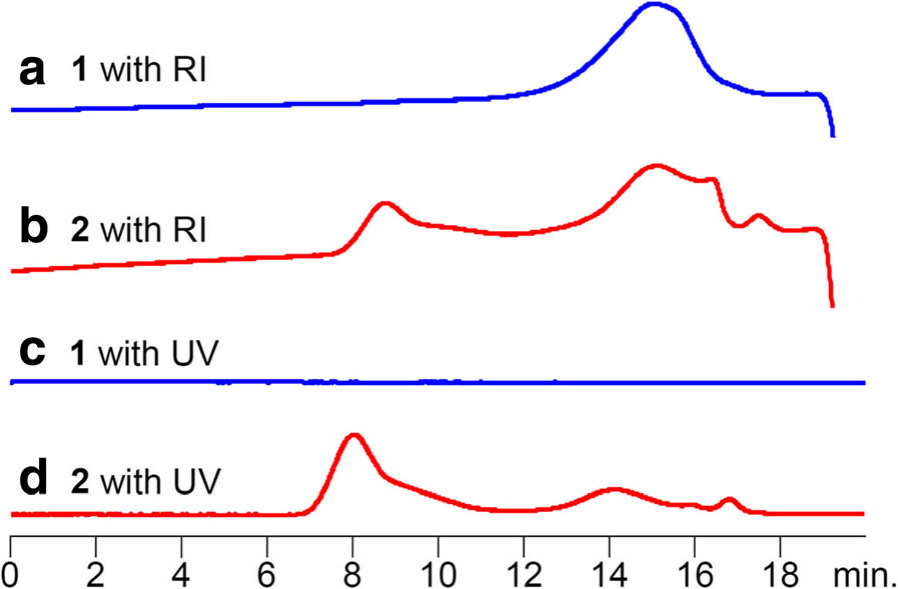
SEC traces of **1** (**a**) and **2** (**b**) with RI detector. SEC traced of **1** (**c**) and **2** (**d**) with UV detector at 500 nm

Figure 2 shows SEC traces of **1** and **2**. The RI trace of **1** has only one broad peak (Fig. 2a); however, the RI trace of **2** has two (Fig. 2b). The peak at around 9 min was not calculated because it was out of range of the standard molecular weight. Additionally, the RI trace of **2** broadened all around. This is probably because **2** includes various comonomer units in copolymers, due to the polymer reaction between **1** and azobenzene. Molecular weights of **1** and **2** shown in Table 1 were determined by SEC with RI detector (Fig. 2a, b). The molecular weight of **2** was calculated from only the low molecular side peak (Fig. 2b, 14.2 min) because the high molecular weight side peak could not be used as it was too high (Fig. 2b, 8.1 min). Generally, the polymer reaction for substituent modification would result in a slight increase of the molecular weight; however, we recognized the separate peak in SEC in the very high molecular weight region (Fig. 2b).

In order to clarify the peaks of **2** as azobenzene units, SEC traces of **1** and **2** were observed using the 500 nm UV absorbance detector (Fig. 2c, d). Naturally, no peak was detected for **1** (Fig. 2c); however, the peaks for **2** were apparent by UV absorbance (Fig. 2d). The difference between **1** and **2** is the presence or absence of the azobenzene units. Therefore, any peaks shown by the UV detector for **2** are derived from azobenzene units. Actually, the RI detector for **2** detected polymers at about 0.5 min after sensing UV absorbance. The peak of **1** at around 15 min detected by RI trace shifted to the low molecular weight side after the polymer reaction (Fig. 2b).

Additionally, the distribution of molecular weight was broadened from 3.8 to 4.8 (Table 1, run 2). This indicates that the introduction ratio of azobenzene units to the copolymer has a wide range. In short, it was estimated that the polymer chain of **2** (*M*_n_ = 4000) possesses four azobenzene units, estimated by SEC and ^1^H NMR. Interestingly, the peak intensity of **2** at around 9 min detected by UV (Fig. 2d) is much larger than that detected by RI (Fig. 2b), when they are compared to the peak of **2** at around 15 min. Taking into account that azobenzene units were only detected by UV absorbance, it suggests that the high molecular weight moiety (Fig. 2d, 8.1 min) is composed of a kind of aggregate enriched by azobenzene, probably due to the π–π interactions [40–42].

**Fig. 3:**
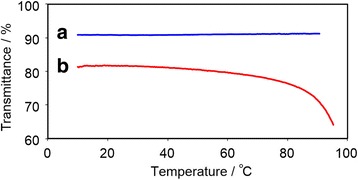
Temperature dependence of light transmittance of 0.2 wt% aqueous solutions of **1** (**a**) and **2** (**b**) in heating processes

Figure 3 shows the light transmittance for aqueous solutions **1** and **2** against the temperature during the heating process. The light transmittance of **1** (hydrophilic copolymer) was constant at all temperatures. In comparison, the light transmittance of **2** declined at high temperature due to hydrophobic interaction derived from the polymer chain and/or azobenzene units. Besides, azobenzene units were attracted not only to hydrophobic interaction but also π–π interaction. In contrast, a 10% difference was observed in the light transmittance of **2** between 20 and 90 °C.

**Fig. 4:**
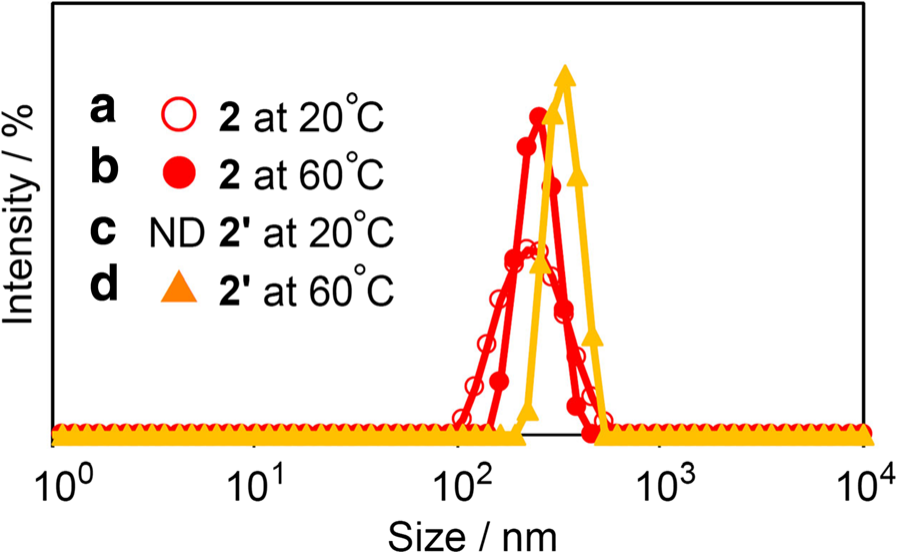
DLS data of 0.2 wt% aqueous solutions of **2** and UV irradiated **2** for 15-min assemblies at different temperatures. **2** at 20 °C (**a**) and at 60 °C (**b**). UV irradiated **2’** at 20 °C (**c**) and 60 °C(**d**)

Figure 4 and Table 2 show DLS data for nanoparticle sizes with 0.2 wt% aqueous solution using **2** and UV irradiated **2** (**2’**) at 20 and 60 °C. The particle sizes of **2** and **2’** were observed: 210 nm(**2** at 20 °C), 250 nm(**2** at 60 °C), and 330 nm(**2’** at 60 °C). The biggest size of assembly was **2’** at 60 °C. For **2’** at 20 °C, the assembly could not be observed because neither polymers were congregated or assembly sizes were off the scale low. The structural isomer of *cis*- and *trans*-configuration of azobenzene might be influential. When azobenzene units become *trans*-configuration, their units were probably attracted by π–π interaction, and therefore, polymers including azobenzene units assembled regardless of the temperature. However, *cis*-configuration azobenzene units were not aggregated possibly because of the lower π–π interaction at low temperature. In addition, both **2** and **2’** peaks of assembly sizes at 60 °C were sharper than the peak of **2** at 20 °C. Probably, assemblies were formed by π–π interaction among azobenzene units and the hydrophobic interaction of polymer chains and azobenzene units at 60 °C Therefore, polymer assemblies were compressed with hydrophobic interaction. Besides, the average sizes of nanostructure **2’** were bigger than **2** at about 80 nm, indicating that assemblies of **2’** were more compressed due to the steric barrier difference of cis-trans structural isomer compared with azobenzene units. On the other hand, assemblies were formed between azobenzene units at 20 °C; then, variability in polymer assembly sizes were observed, such as the broad peak in the DLS data. Assembly sizes of **2** were 40 nm different between 20 and 60 °C which corresponded to the change of light transmittance.

**Table 2:** DLS data of **2** and UV irradiated **2**

Run	Sample	Temperature (°C)	Size (nm)
1	**2**	20	210
2	**2**	60	250
3	**2’** (UV irradiation)	20	ND
4	**2’** (UV irradiation)	60	330

*ND* not determined

## Conclusions

**1** was successfully synthesized by radical polymerization using AIBN after a photoresponsive and thermosensitive polymer of **2** was obtained. Going forward, facile synthesis of stimuli-responsive polymers is achievable and accelerated development of these polymers is anticipated. Above all, a multi-stimuli-polymer that is thermosensitive and photosensitive **2** was aggregated in four-sized assemblies by thermal and UV stimuli in water, which were observed by DLS measurement. We assume two reasons: firstly, the hydrophobic interactions between polymer chains and azobenzene units were strengthened as the temperature was raised. Consequently, the assembly sizes of polymers were changed depending on temperature. Secondly, the chemical structure was differently derived from cis-trans structural isomer of azobenzene units when irradiated by UV. In summary, the π–π interaction between azobenzene units was unlikely to disappear and the effect of steric hindrance was diminished. As a result, the aggregation process was transmuted and assembly sizes were different. Specifically, assemblies could not be observed because neither polymers were congregated, nor assembly sizes were too low at the showing of UV irradiated at low temperature. It is revealed that aggregations of **2** changed to several aggregation structures by UV light and temperature. We are motivated to utilize these aggregated structures and to achieve more sensitive control of the aggregation size.

## Additional File

Additional file 1: Supporting information. (DOCX 343 kb)

## Abbreviations

AIBN: Azobisisobutyronitrile
LCST: Lower critical solution temperature
MOENVF: *N*-Methoxyethyl-*N*-vinylformamide
NVFazo: 4-(2-*N*-Vinylformamide)ethoxyazobenzene
PNVA: Poly(*N*-vinylacetamide)
PNVF: Poly(*N*-vinylformamide)
PNVIBA: Poly(*N*-vinylisobutylamide)
