# Supplementary material for: Aggregation Control by Multi-stimuli-Responsive Poly(N-vinylamide) Derivatives in Aqueous System
Source: Nanoscale Res Lett. 2017 Jul 21;12:461. doi: 10.1186/s11671-017-2221-7 (PMC5520822; doi:10.1186/s11671-017-2221-7)
Supplement: Additional file 1: — Supporting information. (DOCX 343 kb) [file 11671_2017_2221_MOESM1_ESM.docx]

**Additional file 1**

**Aggregation control by multi-stimuli-responsive poly(*N*-vinylamide) derivatives in aqueous system**

Ryo Kawatani, Yasuhiro Nishiyama, Hironari Kamikubo, Kiyomi Kakiuchi, and Hiroharu Ajiro*

**Figure S1.** ^1^H NMR spectrum of MOENVF in CDCl_3_ (400MHz, r.t.).

**Figure S2.** ^1^H NMR spectrum of **1** in D_2_O (400MHz, r.t.).

**Figure S3.** ^1^H NMR spectrum of **2** in DMSO-*d*_6_ (400MHz, r.t.).


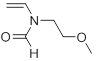

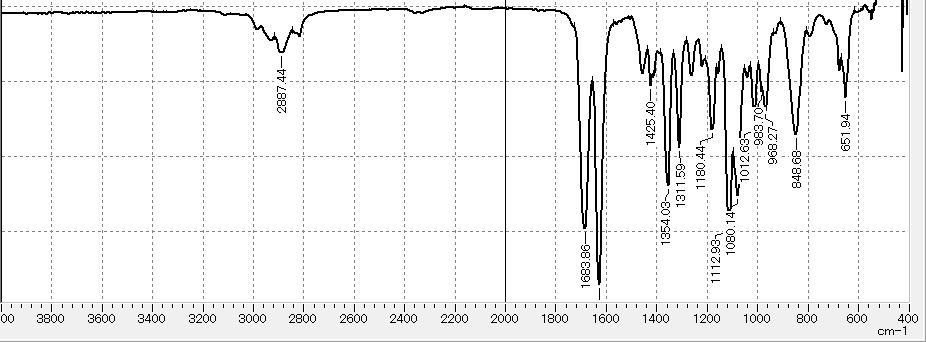


cm^-1^

**Figure S4.** IR spectrum of MOENVF.


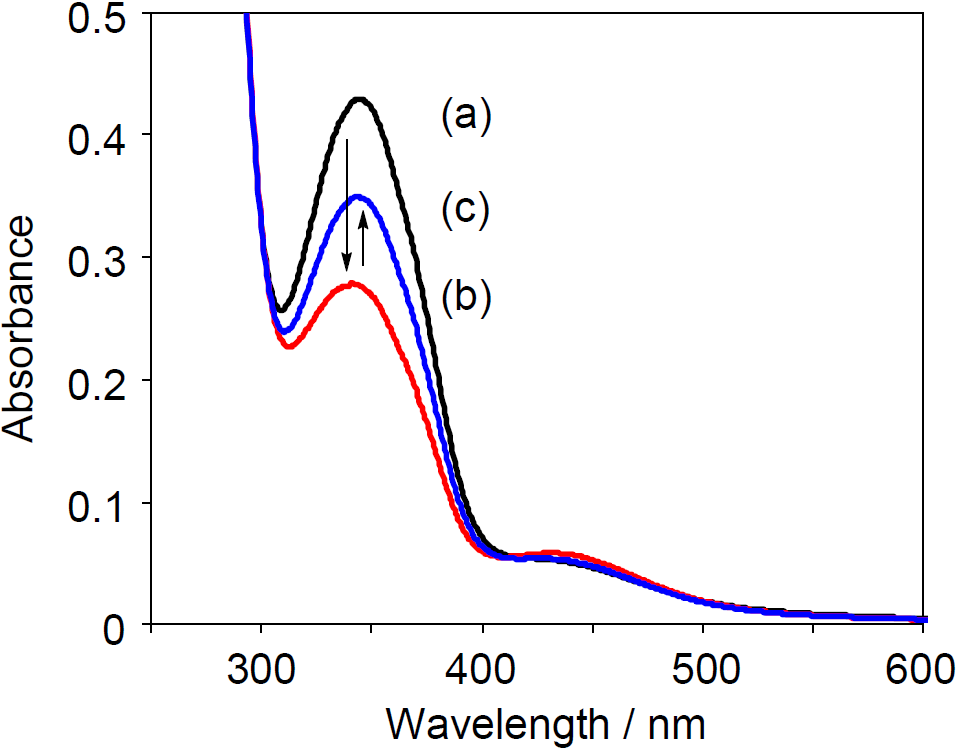


**Figure S5.** UV spectra of **2** (a), after UV light (330 nm) irradiation for 10 min. (b), and the successive visible light irradiation for 5 min. (c). These UV spectra were monitored by V-630 (JASCO Corporation).
